# The edge visualization metric: Quantifying the improvement of lung SBRT target definition with 4D CBCT

**DOI:** 10.1002/acm2.70114

**Published:** 2025-06-05

**Authors:** Colton Baley, Sam Andersen, Clark Anderson, Shraddha Dalwadi, Daniel L. Saenz

**Affiliations:** ^1^ Department of Radiation Oncology Mays Cancer Center at UT Health San Antonio San Antonio Texas USA

**Keywords:** CBCT, lung, radiation therapy, SBRT

## Abstract

**Purpose:**

Four‐dimensional cone‐beam CT (4D CBCT) incorporates oversampling of 3D data to reconstruct multi‐phase CBCT data sets representing distinct phases of the breathing cycle based on a diaphragmatic correlate of respiratory motion. Motion artifacts and blurring can be reduced relative to three‐dimensional cone‐beam (3D CBCT), allowing clinicians to better visualize motion of targets. To quantitatively understand the degree to which target visualization is improved by 4D CBCT, an edge visualization metric (EVM) has been developed to describe the change in voxel intensities at the edge of targets in 4D CBCT maximum intensity projection images relative to 3D CBCT images.

**Methods:**

The EVM describes the median distance where voxel intensities drop from 80% to 20% of target voxel values. The EVM was evaluated in a phantom study with a CIRS dynamic thorax phantom and with eleven on‐treatment lung SBRT patients.

**Results:**

In the phantom study, the EVM was improved for 4D CBCT relative to 3D CBCT for one‐cm targets (2.43 ± 0.22 mm vs. 2.67 ± 0.31 mm, *p* = 0.04) and for 2‐cm targets (2.60 ± 0.35 mm vs. 3.46 ± 1.03 mm, *p* = 0.02). In patients, the EVM was 3.59 ± 1.01 mm vs. 4.25 ± 1.24 mm (*p* < 0.05).

**Conclusions:**

When evaluating an imaging acquisition's degree of motion blurring and ability to delineate target edges, EVM may provide a less biased way to evaluate edge detection in the presence of motion when compared to traditional methods.

## 1 INTRODUCTION

Modern management of cancer and other indications with therapeutic radiation regularly incorporates image‐guided radiation therapy (IGRT) for precise target localization during treatment. Such image‐guidance is critical for high precision techniques such as stereotactic body radiation therapy (SBRT), which utilizes high dose per fraction and limited beam margins due to increased setup accuracy allowed by improved immobilization as well as high confidence in imaging. SBRT is particularly useful in non‐small cell lung cancer, where three‐year control rates reach 70%–90% (resulting in survival rates of 50%–70%).^1^ Computed tomography (CT) serves as the prevailing standard across most treatment sites for simulation, particularly relying on three‐dimensional cone beam computed tomography (3D CBCT) at the time of treatment.^2^, ^3^ However, in areas of the body prone to significant motion, such as the lungs, 3D CBCT often presents notable motion artifacts. These artifacts distort pixel values along the target envelope boundary, inaccurately representing the full scope of target motion. In contrast, four‐dimensional CT (4D CT) at simulation incorporates a temporal element into imaging acquisition, enhancing resolution of the tumor envelope and leading to better internal target volume (ITV) delineation. 4D CT‐based planning has become widespread for mobile lung targets, and the 4D CT serves as the reference image during IGRT.

During IGRT, however, the motion artifacts and reduction in grayscale fidelity along the target‐envelope boundary impacts the registration of the 3D CBCT with the reference 4D CT image. As a result, there is an increase in both systematic and random error in image matching, culminating in the necessity for a larger planning target volume. These errors may be mitigated by improving the visualization of the target envelope's edges in the treatment imaging.

The ability to sufficiently determine the target boundary during treatment may be achieved using Four‐dimensional cone‐beam CT (4D CBCT), which introduces a temporal element into 3D CBCT.^4^, ^5^ Previous studies have shown that 4D CBCT is less affected by motion artifacts and severe blurring in the presence of respiratory motion when compared to 3D CBCT.^6^ Moreover, objective assessments using phantom data have revealed that 3D CBCT exhibits inferior image quality when evaluated through metrics such as contrast transfer function, noise power spectrum, and modulation transfer function. Conversely, image quality in patient studies is more difficult to assess, but 4D CBCT has demonstrated the ability to improve the accuracy of image‐guidance using observers to subjectively score images.^7^ Additionally, 4D CBCT has been shown to measure mobile target trajectory similarly to 4D CT.^8^ While several potential benefits of 4D CBCT are illustrated in these studies, a quantitative evaluation of the benefit provided by 4D CBCT is important to objectively demonstrate improvement. A method to more directly measure target edge delineation in patient data using 4D CBCT would therefore be of great interest, not only in quantitatively evaluating better edge resolution of 4D CBCT, but also as a potential benchmark of in vivo 4D CBCT imaging quality for future studies.

The ability to delineate an edge is of course complex, as it relates to how a target motion envelope(ITV) is resolved from the point of view of automatic image registration tools or the human eye reviewing an image. Delineation of an edge will involve detecting its location as well as its sharpness. Edge detection is a well‐established image processing tool in medical imaging.^9^, ^10^ Common tools include gradient‐based^11^ methods as well as methods relying on Gaussian filters.^12^ Such methods are candidates for edge detection, which could then be used to potentially define how sharp the edge is. Other methods include the edge spread function (ESF).^13^ The ESF is used to estimate the modulation transfer function as a metric of imaging system performance. However, measurement near an edge can be difficult due to the inhomogeneity of Hounsfield units near edges, and also depends on the accuracy of the phase alignment in the ESF sample.^14^ Nevertheless, HU variations in lung and pelvis CBCT have been reduced by <100 HU by correcting for scatter with ESF. The clinical detection of an edge will also be complicated by the surrounding anatomy. Lung targets surrounded only by lung are easier to visualize. However, surrounding lung vessels, for instance, could reduce the ability to see the edge of the motion envelope.

In this study, we will utilize both phantom and patient 3D CBCT and 4D CBCT data to develop a metric that quantitatively evaluates the degree to which mobile target edges in patient data may be more precisely delineated in 4D CBCT when compared to 3D CBCT. The metric will be built to be robust to the presence of varied surrounding anatomy. We will use patient‐derived motion to conduct the phantom studies. The EVM magnitude in 3D and 4D CBCT will be examined to test the hypothesis that 4D CBCT will provide sharper edges (lower EVM values). From the results, clinical applications will be proposed as they relate to CBCT protocol optimization and cost‐benefit analysis for image quality versus image dose.

## 2 METHODS

### 2.1 Imaging data sources

4D‐CBCT imaging was performed with both phantom data and patient data. Phantom data was acquired using the Computerized Imaging Reference Systems (CIRS, Norfolk, VA, USA) dynamic thorax phantom model 008A. Targets used for the study included 1‐cm and 2‐cm diameter soft tissue equivalent spheres. Respiratory waveform data acquired from an external surrogate tracking point during the 4DCT study from seven previously treated patients were used to create patient‐specific waveforms. The amplitude traces were smoothed with a Savitzky‐Golay filter. The average peak‐to‐trough distance in the external surrogate was scaled to the 4D‐CT motion extent in all the three directions separately. This methodology has also been previously described in an earlier published work.^8^

Patient data was acquired for 11 patients undergoing lung SBRT or hypo‐fractionated therapy with less than 10 fractions using an SBRT simulation setup. These patients were enrolled in an institutional review board approved study. Eight patients had targets in the lower lobe of the lung and three were in the upper lobe. Six patients had left‐sided disease, while five had right‐sided disease.

### 2.2 Imaging methods

4D‐CBCT imaging was conducted on an Elekta VersaHD linear accelerator with a Symmetry VolumeView study on the XVI system (Elekta, Stockholm, Sweden). The protocol consisted of 975 frames, each with 20 mA and 16 ms per frame. The scan covered a 200‐degree counterclockwise arc of gantry motion, and the side of rotation depended on the location of the target (4 min scan time). The Elekta F0 filter and S20 collimator were employed. Image reconstruction utilized a pixel spacing and nominal slice thickness of 2 mm. Additionally, a 3D CBCT was acquired for each patient. This 3D CBCT scanning protocol consisted of 200 frames (20 mA and 10 ms per frame) over approximately a 200‐degree rotation (70 s scan time). Depending on the arc length, the rotation may have been slightly larger. The F0 filter and S20 collimator were used. All patients underwent free breathing during both 3D‐ and 4D‐CBCT imaging. All images were exported at a slice thickness and resolution of 1 mm for analysis. Two physicians contoured the target motion envelope (ITV) on a maximum intensity projection (MIP) of all phases of the 4D‐CBCT as well as on the 3D‐CBCT.

### 2.3 Image processing

The 4D CBCT MIP was used for 4D CBCT evaluation as it represents the union of all 10 phases displayed at the treatment console during IGRT evaluation. Both 3D and 4D data sets expressed in HU were then filtered with a 5 × 5 median filter, which is a non‐linear filter that removes noisy pixels while preserving image features such as edges.^15^

### 2.4 Edge measurement

**FIGURE 1:**
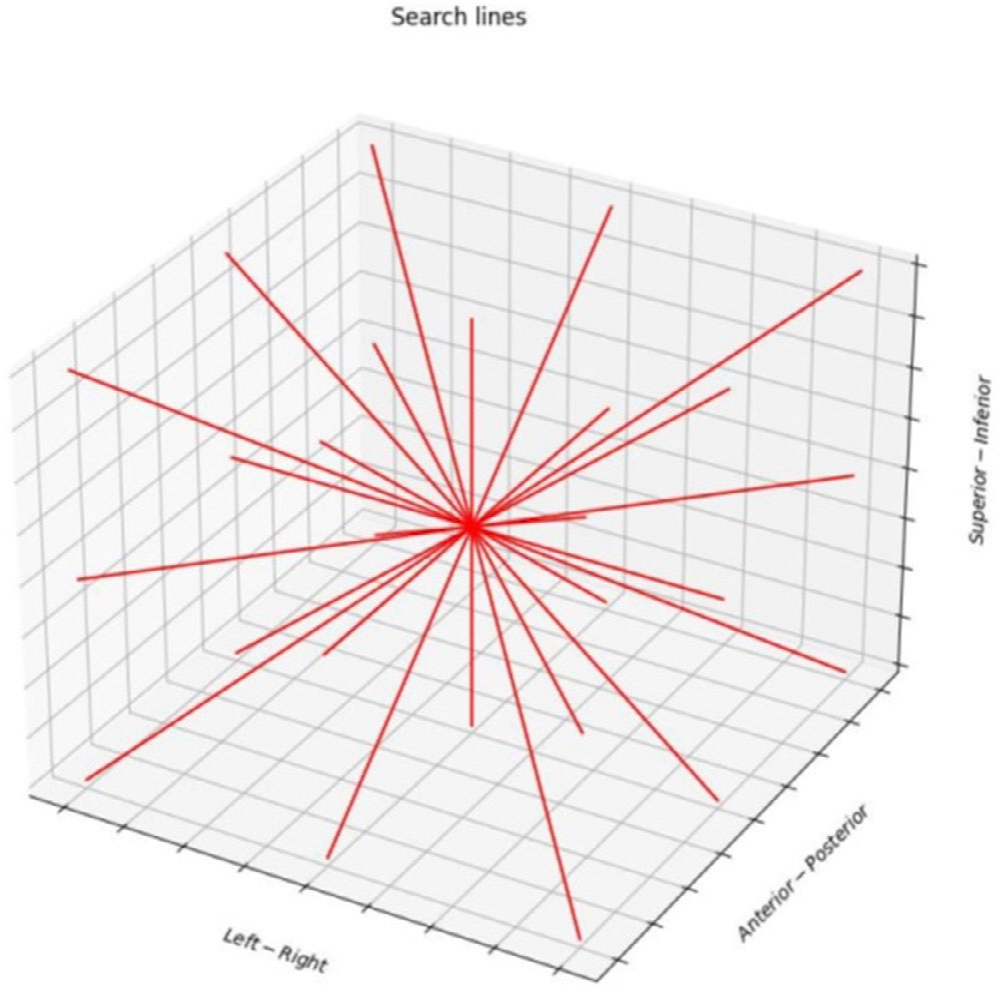
A visual representation of the 26‐line search directions implemented for the EVM. Direction included all 3D space 45‐degree angle combinations of SI, AP, and LR. For example, line directions would be right, right/anterior, right/superior, right/anterior/superior and so forth. All lines started at the same origin being the approximate target centroid. EVM, edge visualization metric.

An in‐house python script was written to develop the edge visualization metric (EVM). Upon filtering image data sets, various line rays were used to find the distance between 80% and 20% target image intensity with respect to the background to quantify the blur of the target edge delineation. Background and target intensities were locally determined along the line rays. Local intensities along the line rays were used instead of a global target and background value to increase the stability and reproducibility of results. To implement this, a line ray was iterated along a pixel‐by‐pixel path, creating an array of values. This line iteration was repeated for all combinations of superior/inferior, anterior/posterior, and left/right directions, leading to every 45‐degree angle in 3D space being explored. The result was a total of 26 lines. While so many lines may be computationally intensive, the clinical application of this metric relates more to upfront CBCT protocol commissioning rather than routine use, so the computation time is not a significant issue. All lines originated from the target centroid determined using coordinates from the physician's target contour. Target centroids were unique for 3D and 4D CBCT data sets. Figure 1 shows a visual representation of all line search directions. Line length was dependent on the direction being searched. Target dimensions were measured from the physician contour in the superior‐inferior (SI), anterior‐posterior (AP), and left‐right (LR) directions. Depending on the search direction, the largest of the dimensions for that search direction was used so that approximately half the values would be inside the target with the remainder outside the target. For example, if the line searched was in the right/superior direction and SI dimension was larger than LR diameter, then SI dimension was chosen as the search length. Once an array of the image intensity values along the line was collected, a histogram was created from the values. The number of bins was determined by taking the square root of the array length, rounded up to the nearest integer. The mean of values in the lowest and highest bins was taken to represent background and target, respectively.

**FIGURE 2:**
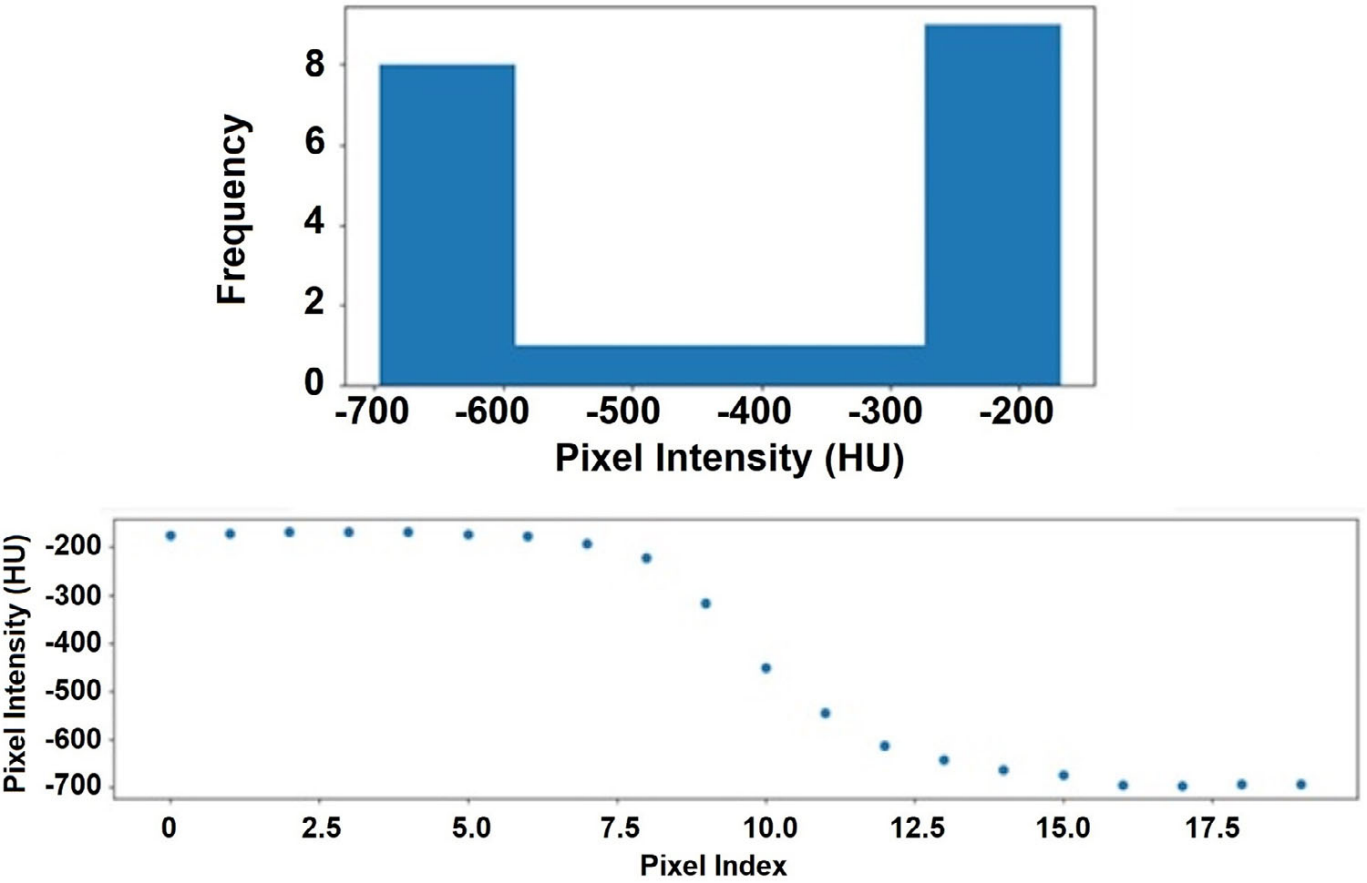
An illustration of the histogram produced from pixel intensities in a search direction. Pixel values are expressed in HU. As can be seen, most values are either in the highest or lowest bin, representing target and background pixel intestines respectively. The bottom scatter plot shows pixel intensity as a function of pixel index or search distance from the origin.

**FIGURE 3:**
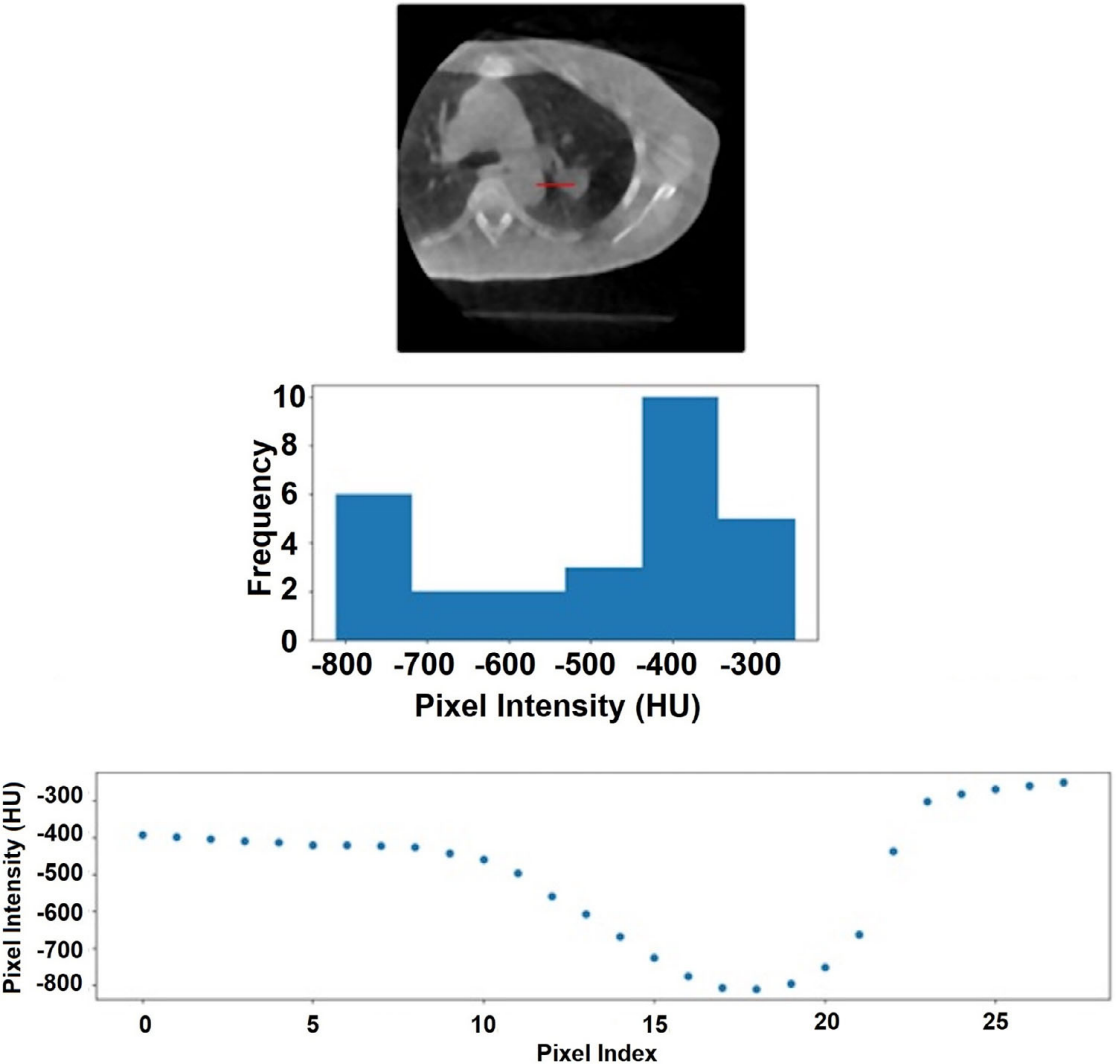
A scenario is shown where the second‐highest histogram bin was used for the target value intensity. As can be seen in the top 3D CBCT axial slice, the line searched in the right direction is traversing through a large object whose intensity is greater than that of the target. This is also demonstrated in the scatter plot on the bottom. The first bin is comprised of values whose indices are exceeding the background indices, which cannot be the case given the origin of the line searching is the target centroid. 3D CBCT, three‐dimensional cone‐beam CT.

Figure 2 shows an example of a histogram and scatter plot of pixel values as a function of line search distance. If the mean index of values in the highest bin was higher than the mean index of values in the lowest bin, the second‐highest bin was used instead. This was implemented to remove faulty target values. Target index values cannot be beyond background, given line iteration starts at the target centroid. If the mean target index values are beyond the target, a large structure outside the target must have been entered. An example scenario where the second bin was used is shown in Figure 3. Once background and target values were calculated, pixel intensities representing 80 and 20% of the mean target intensity along the search path were determined with the following equations:

$$80\%\mathrm{value}=\mathrm{Background}+0.8\times \left(\mathrm{Target}-\mathrm{Background}\right)$$

$$20\%\mathrm{value}=\mathrm{Background}+0.2\times \left(\mathrm{Target}-\mathrm{Background}\right)$$

where background and target are the mean values determined from the histogram of line search array values. Since we are interested in the sharpness of the visualized edge of the target, this is quantitatively similar to the penumbra in a radiation beam. Hence, we adapted the well‐established 80% to 20% metric used for that analysis. The array of values from the line search were then iterated through to find the pixel index that first reaches the 80% value. Since the 80% value is likely between two pixels, the pixel index value was then interpolated. The process was repeated for the 20% value. The Euclidean distance between 80‐ and 20%‐pixel indices was then converted to distance in millimeters by factoring in slice thickness and resolution. This distance over all 26 directions searched was used as the basis for EVM.

**FIGURE 4:**
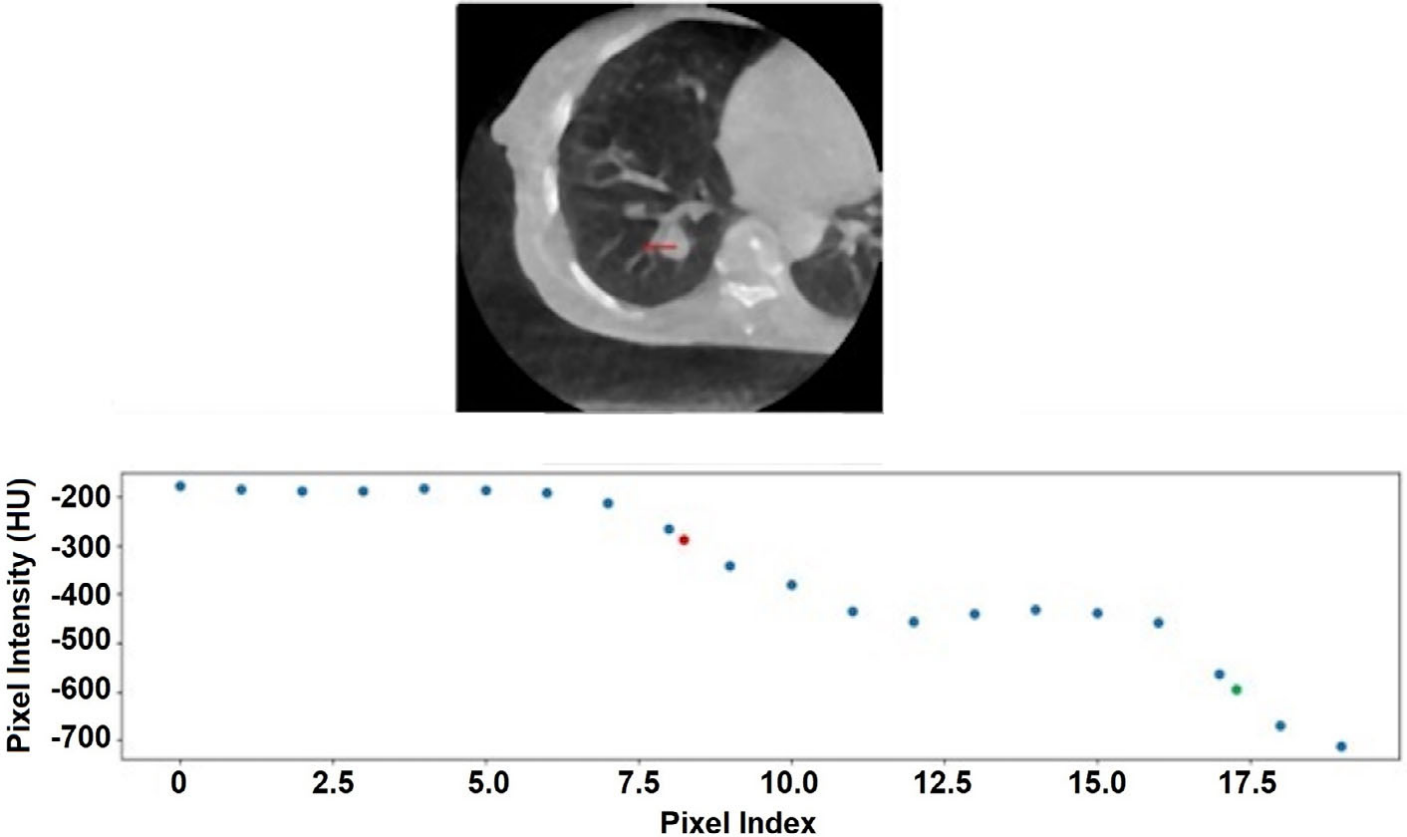
Example of a change in direction of the slope of pixel intensities. The red dot on the scatter plot represents the interpolated 80% of target intensity value. Similarly, the green dot represents the interpolated 20% of target intensity value. As can be seen on the scatter plot, the decrease in intensities plateaus and increases slightly for several pixels before finding the 20% value. This alludes to passage through a nearby structure as seen in the 4D CBCT MIP axial slice above the scatter plot. The line search in the right direction is passing through a large vessel. 4D CBCT, Four‐dimensional cone‐beam CT; MIP, maximum intensity projection.

**FIGURE 5:**
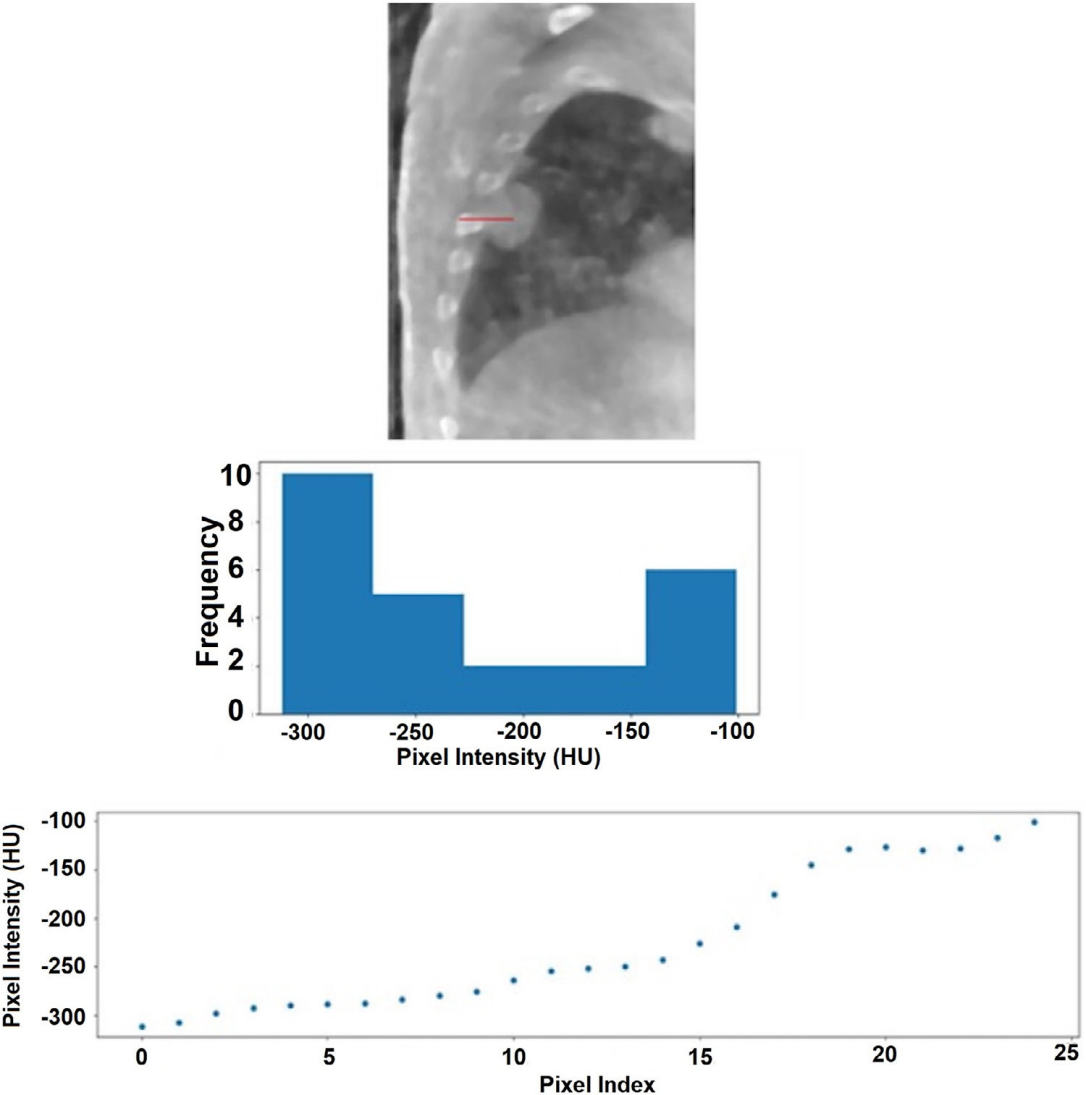
The top image is a sagittal slice from a 4D CBCT MIP. The posterior ray is traversing through a rib. The effects on line search values are shown in the histogram and scatter plots. As can be seen, the line starts in the target, but the pixel intensities only increase, making this line difficult to analyze with the current methodology. These scenarios were automatically thrown out due to pixel values increasing excessively. 4D CBCT, Four‐dimensional cone‐beam CT; MIP, maximum intensity projection.

**FIGURE 6:**
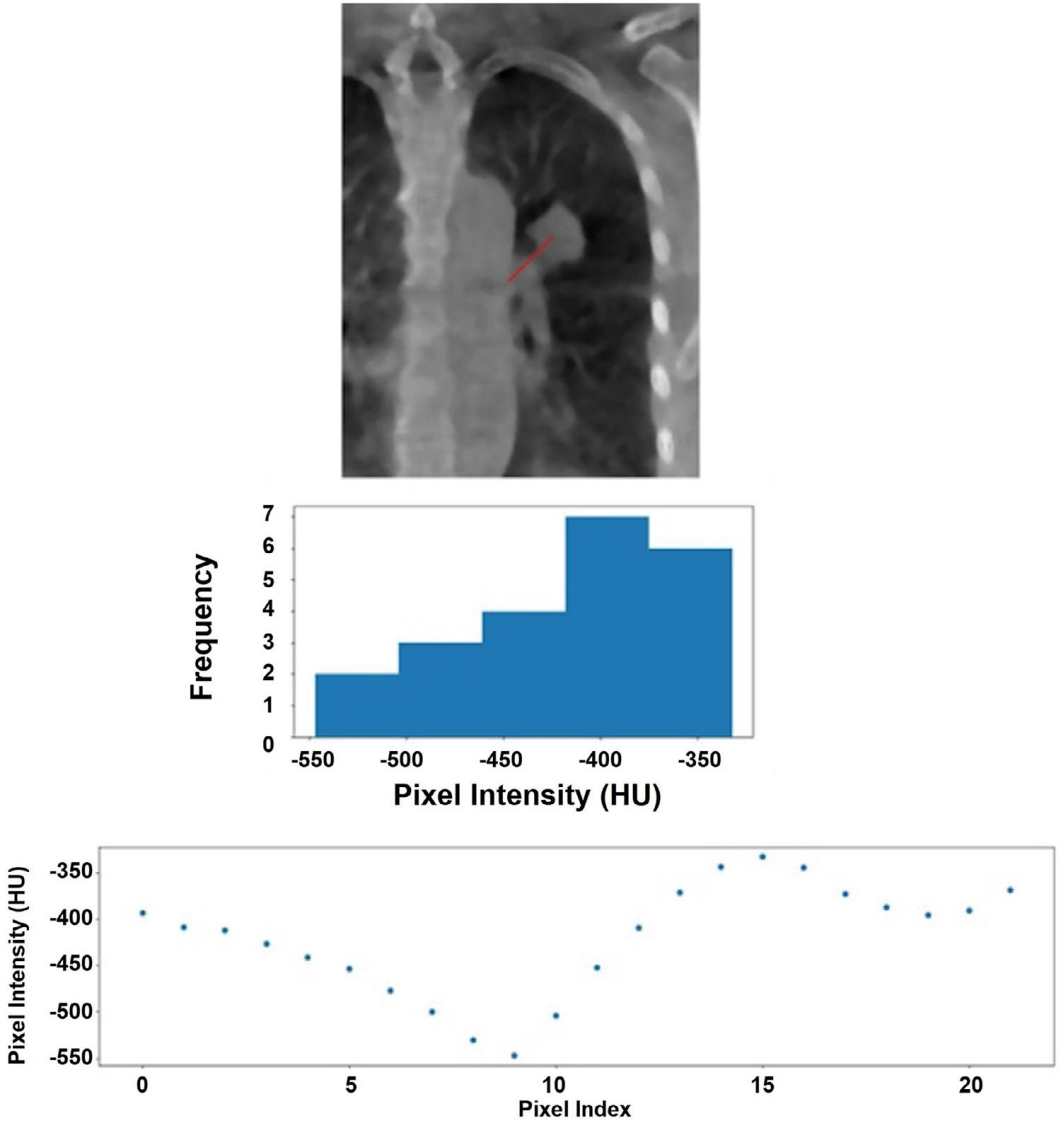
An example of the two highest pixel intensity two bins not accurately representing the target value. The mean index for the two bins is greater than lowest bins index. Additionally, the ray in this case never really leaves soft tissue. The top image is a 3D CBCT coronal slice of the ray being searched to create the histogram and scatter plot. 3D CBCT, three‐dimensional cone‐beam CT.

Additional criteria were added to increase the stability of the EVM. If slope changed direction between the search for 80%‐ and 20%‐pixel indices, the value from that direction was not considered. This was to remove distance values that may have been excessively large due to entering a structure after finding the 80% index but before finding the 20% index. An example of a slope change is shown in Figure 4. Similarly, if the line search values began to increase excessively, the iteration was stopped since the search was likely going through bone. A posterior line going through bone is illustrated in Figure 5. Lastly, if the first and second bin had a mean index greater than that of the background bin's mean index, this value was excluded from the results as well. After the first and second bin, there was too much uncertainty in automatically determining which bin accurately represented the target value. Figure 6 demonstrates the uncertainty after ruling out the first two bins. All exclusion criteria were built into the script, and removal of data points was automatic.

The EVM was based on the median of the results from all directions. The median was chosen so that, in the event outlier values from a few directions were included, the descriptive statistic would not be significantly influenced.

### 2.5 Statistical analysis

EVM data were evaluated first for whether they followed a normal distribution. A Shapiro–Wilk test was conducted to determine if there is a significant departure from normality. Additionally, results were plotted using a histogram to inspect normality. If the data could be characterized by a normal distribution, a student's paired *t*‐test was utilized to evaluate if the distributions were significantly different. If data showed a departure from normality, the non‐parametric Wilcoxon signed‐rank was utilized.

## 3 RESULTS

### 3.1 Phantom imaging

In the phantom imaging, no values needed to be removed due to the homogeneity of the phantom. Table 1 shows EVM values for each patient waveform analyzed for both target sizes. The average median value and standard deviation (SD) for the 1‐cm target size for 3D and 4D CBCT MIP were 2.55 ± 0.26 and 2.32 ± 0.24 mm, respectively. The phantom EVM data did not show a significant departure from normality (*p* = 0.3013, *p* = 0.6841, *p* = 0.6782, *p* = 0.1759 for the respective columns in Table 1). With a paired *t*‐test, the difference in EVM was significant (*p* = 0.01). Similarly, the 2‐cm target size average median value and SD were 3.29 ± 0.96 and 2.55 ± 0.33 mm for 3D and 4D CBCT MIP, respectively. The difference in EVM for the 2‐cm target was significant (*p* = 0.05).

**TABLE 1:** Tabulation of phantom EVM results for 3D and 4D CBCT MIP.

Patient	1 cm		2 cm	
	3D CBCT (mm)	4D CBCT (mm)	3D CBCT (mm)	4D CBCT (mm)
1	2.88	2.69	4.04	3.22
2	2.30	2.15	2.12	2.23
3	2.67	2.24	4.28	2.33
4	2.73	2.45	4.89	2.60
5	2.47	2.45	3.47	2.55
6	2.63	2.41	2.83	2.91
7	2.03	2.03	2.09	2.22
8	2.66	2.48	2.99	2.58
9	2.61	1.97	2.89	2.33
**Mean ± SD**	2.55 ± 0.26	2.32 ± 0.24	3.29 ± 0.96	2.55 ± 0.33

*Note* Target sizes of 1 and 2 cm diameter were evaluated for nine patient‐specific waveforms. Mean EVM and one SD are shown in the bottom row.

Abbreviations: 3D CBCT, three‐dimensional cone‐beam CT; 4D CBCT, Four‐dimensional cone‐beam CT; EVM, edge visualization metric; MIP, maximum intensity projection; SD, standard deviation.

### 3.2 Patient imaging

Table 2 shows the EVM values for the patient imaging for both 3D and 4D CBCT, as well as the number of points removed. For the eleven patients enrolled in the study who received both 4D CBCT and 3D CBCT imaging, the average median values were 4.25 ± 1.24 and 3.59 ± 1.01 mm for 3D and 4D CBCT MIP, respectively. The Shapiro–Wilk test did demonstrate a departure from normality for 4D‐CBCT data (*p* = 0.0258) while the 3D CBCT data did not (*p* = 0.1977). The Wilcoxon signed‐rank test indicated a *W* value of 9, where the critical value for *n *= 11 was 11, meaning the result was statistically significant with a *p *< 0.05. Stratification was applied (Table 3) based on lobe of the lung (*n* = 3 for upper and *n* = 8 for lower). There were no patients with targets in the middle lobe. No statistical analysis was performed with lobe stratification due to low sample size. However, mean and SD in EVM were increased in the lower lobes, reflecting the expected increased target motion in the lower lobes.

**TABLE 2:** Results of EVM for all patients.

Patient	EVM (mm)		Points removed	
	3D CBCT (mm)	4D CBCT (mm)	3D CBCT	4D CBCT
1	3.84	2.76	1	2
2	4.60	4.40	5	4
3	2.89	3.15	5	3
4	3.78	2.57	2	3
5	7.11	4.94	11	11
6	2.92	2.95	1	0
7	3.77	2.97	14	16
8	4.89	3.35	2	4
9	3.2	2.5	7	5
10	5.34	4.93	2	6
11	4.44	4.99	7	10
**Mean ± SD**	4.25 ± 1.24	3.59 ± 1.01	–	–

*Note* The number of points removed via exclusion criteria are also shown in the two right columns. Mean EVM and one SD are provided in the bottom row.

**^Abbreviations:^ :**3D CBCT, three‐dimensional cone‐beam CT; 4D CBCT, Four‐dimensional cone‐beam CT; EVM, edge visualization metric; SD, standard deviation.

**TABLE 3:** Results of EVM (mean and SD) stratified by upper and lower lobes.

	EVM Mean ± SD (mm)	
Lobe	3D CBCT	4D CBCT
**Upper (*n* = 3)**	3.20 ± 0.5	2.89 ± 0.29
**Lower (*n* = 8)**	4.65 ± 1.20	3.85 ± 1.07

Abbreviations: 3D CBCT, three‐dimensional cone‐beam CT; 4D CBCT, Four‐dimensional cone‐beam CT; EVM, edge visualization metric; SD, standard deviation.

## 4 DISCUSSION

The concern of motion artifacts in 3D CBCT is well understood and abated by implementation of 4D CBCT. Most current objective metrics comparing image quality between two imaging modalities requires imaging of specialized phantoms.^6^ Additionally, current metrics to evaluate image quality in patient data are highly subjective based on metrics such as observer scoring.^16^ The intention of this study was to provide a more objective measure of image degradation due to target blurring from respiration as it is manifested through object edges. Specifically, the goal was to employ the EVM in patient data to discern the difference in target blurring between 3D and 4D CBCT in a more objective manner than observer scoring.

The EVM in phantom data was significantly worse for 3D CBCT versus 4D CBCT MIP for both 1‐cm and 2‐cm diameter target sizes (*p* = 0.01 and *p* = 0.05, respectively). Additionally, as target size grew, the mean difference in EVM between 3D and 4D CBCT MIP appeared to increase (0.23 and 0.74 mm, respectively). This may be since 4D CBCT MIP remained relatively stable while 3D CBCT results seem to have gotten worse as target size increased. The increase in EVM values with respect to target size for 3D CBCT may be due to the gross increase in motion. For example, if the programmed amplitude was 5 mm, the total extent of target envelope would be 20 and 30 mm for 1 and 2 cm diameters, respectively. The increased range likely causes more blurring in 3D CBCT. The stability of EVM results between target sizes further illustrates that 4D CBCT MIP is better able to resolve targets when compared to 3D CBCT. Patient data mean EVM differences between the two imaging modalities were significant and 0.66 mm higher in 3D relative to 4D CBCT. This mean difference in EVM happened to match the 2‐cm phantom mean difference as well.

A reduction in EVM in patients from 4.25 to 3.59 mm represents an important finding, because it provides quantitative evidence that the edges of targets are sharper in 4D CBCT versus 3D CBCT. A more rapid change in pixel intensity values indicates better visualization of the edge of a target as well as less blurring due to motion artifacts. Better certainty in the target edge can result in higher confidence in targeting and potentially lower setup margins in the future.

Regarding clinical application, the EVM can be used to benchmark 4D CBCT acquisition protocols as it relates to their ability to suppress motion artifacts. For instance, baseline EVM values could be acquired for a certain motion phantom which could be compared with EVM values taken after the adjustment of gantry speed, number of frames, and so forth, in a 4D CBCT protocol. The metric could also be evaluated as a function of total imaging dose to optimize the value of 4D CBCT imaging, which can represent a significant imaging dose. Finally, it can serve as a metric during CBCT protocol optimization, where the kV, ms, gantry rotation speed, and number of frames could be varied to find the combination of parameters producing the best visualized mobile target edges.

**FIGURE 7:**
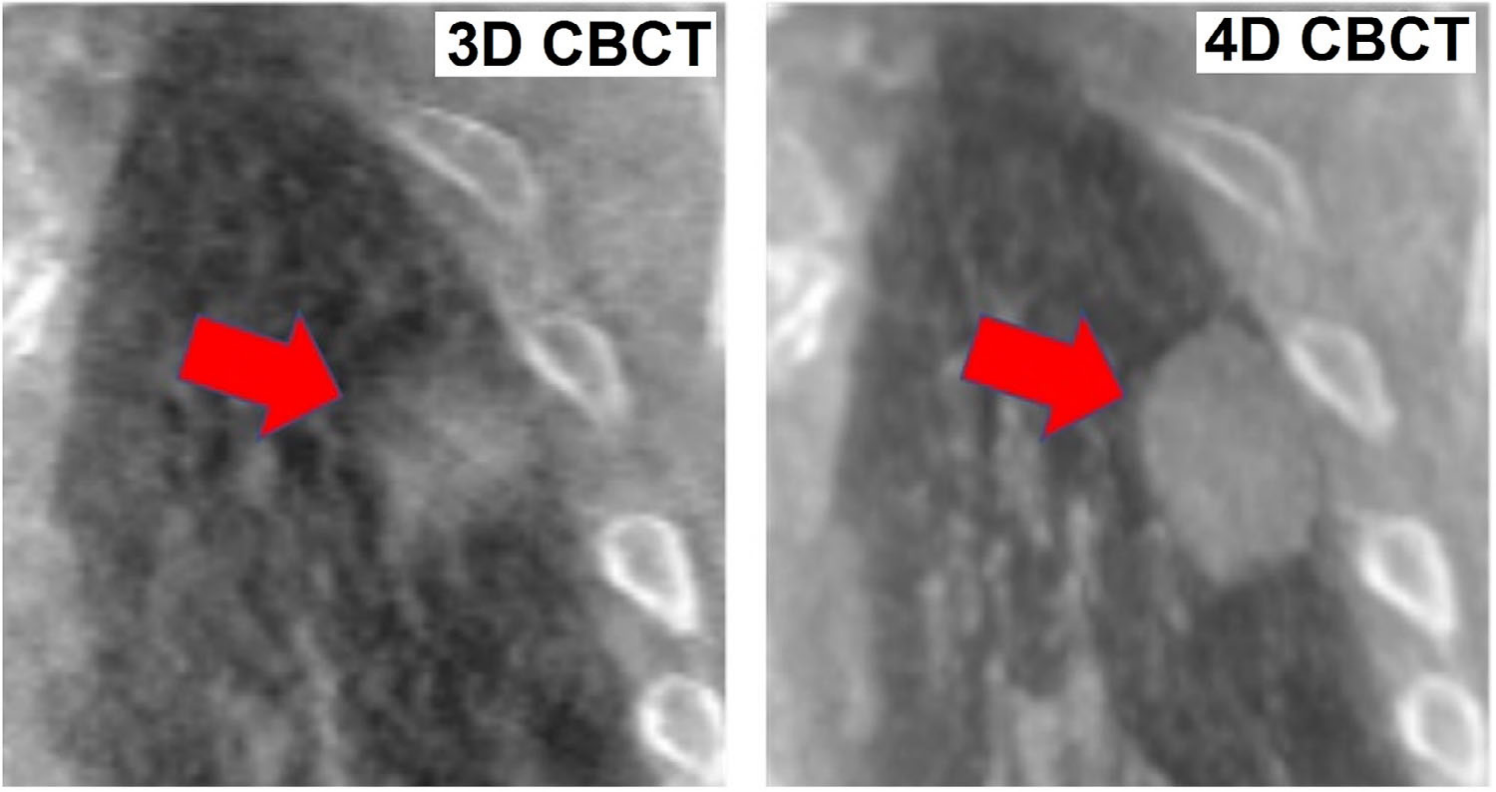
Sample subject for whom the difference between 4D CBCT and 3D CBCT was more pronounced. The EVM difference was 2.2 mm, and the degree of motion was 1.5 cm (for a 2 cm target). 3D CBCT, three‐dimensional cone‐beam CT; 4D CBCT, Four‐dimensional cone‐beam CT; EVM, edge visualization metric.

**FIGURE 8:**
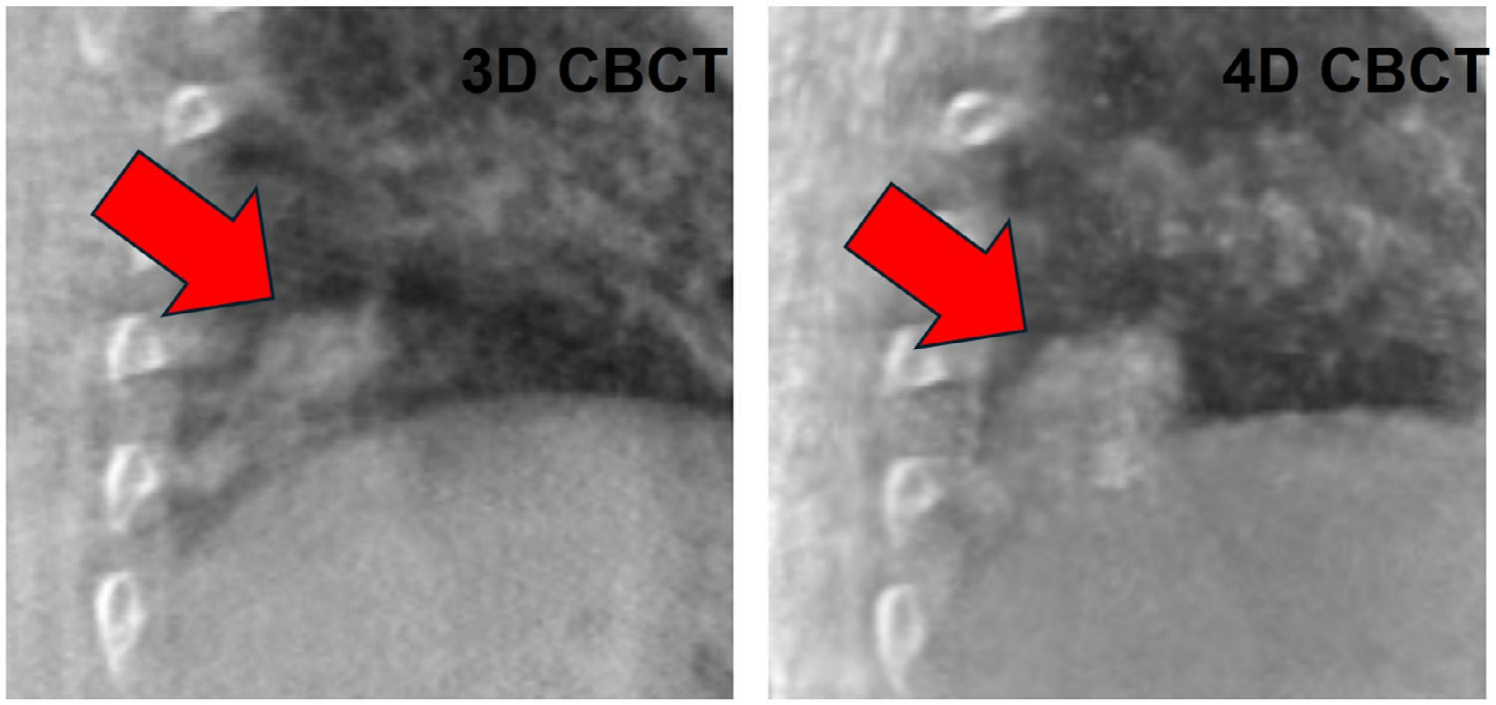
Another sample subject exemplifying increased sharpness and motion trajectory determination near the diaphragm.

Although on average the EVM was improved by 0.66 mm with 4D CBCT, not all patients exhibit the same amount of improvement. For instance, five subjects showed about the same EVM value between both types of imaging (EVM differing by less than 0.5 mm). However, one subject had an EVM of 7.1 mm on 3D CBCT versus 4.9 mm on 4D CBCT, a reduction of 2.2 mm. The sample coronal slice shown in Figure 7 indicates a case when the amplitude of motion was larger (1.5 cm total motion for a 2 cm target size). Future work might focus on the specification of patients for whom 4D CBCT may be the most useful. Figure 8 also exemplifies increased sharpness with target motion.

One limitation of the phantom study was the exclusion of AP and LR motion. AP and LR motion were not explored since previous research has shown that motion is most significant in amplitude in the SI direction.^17^ This is also supported by the patient waveform evaluations, which had SI, AP, and LR motion. While motion was only superior‐inferior for phantoms, patient data obviously included motion in all three directions. For patient EVM evaluation, the primary limitation was sample size. Additionally, with a small sample, the effects of target size or lung lobe location could not be concluded. It stands to reason since phantom data demonstrated a significant difference using a homogeneous phantom with the traditional 3D CBCT protocol, introducing heterogenous patient data may favor 4D CBCT even further, despite the use of 3D CBCT acquisition. Lastly, the origin of line search was based on the centroid of target contours. Of course, the centroid of contours won't reflect all the variation from inter‐observer contours and may in fact be relatively robust to it. This nevertheless, introduces the subjectivity of contouring into results but was seen as the most consistent and accurate way to establish a starting point. While this may introduce some subjectivity, it is small compared to the objectivity resulting from standard 80% to 20% distance measurement introduced by EVM. In fact, with the highly adaptable nature of EVM to be sensitive to local variations in image pixel values both inside and outside of the target and to be flexible with respect to expected anatomical complications in lung CBCT, this method is far more well‐suited to be used objectively than a more generally applied tool such as ESF, which can be sensitive to image homogeneity as well as ROI‐placement.

Finally, since 3D CBCT is known to struggle with motion artifacts, 3D CBCT may have disproportionately been affected by contours, leading to less accurate centroids and thus starting points.

## 5 CONCLUSIONS

The EVM was developed to evaluate target edge detection between 3D and 4D CBCT MIP imaging acquisitions in the context of respiratory motion. The EVM showed statistically significant differences between 3D and 4D CBCT imaging using a dynamic respiratory phantom with 1 and 2 cm target diameters. Additionally, 4D CBCT MIP results appeared to be more stable as the target size increased. Patient data sets demonstrated a difference between the two imaging modalities, as well as a statistically significant reduction in EVM for 4D CBCT. When evaluating the degree of motion blurring for an image acquisition and the ability to delineate target edges in vivo, EVM may provide a less biased way to evaluate edge detection in the presence of target motion when compared to traditional methods.

## AUTHOR CONTRIBUTIONS

Dr Colton Baley contributed to the idea generation (in collaboration with Dr Daniel L Saenz), the hypothesis generation, the data collection and processing of phantom data as well as the processing of patient data, the software design to implement EVM, as well as half of the writing (specifically introduction and methods sections). He reviewed and approved the final manuscript. Drs. Andersen and Anderson both contributed to the target contouring as physicians. They also participated in data acquisition regarding some of the clinical details needed for analysis. In the written manuscript, they reviewed and provided commentary and critique of the final language. They reviewed and approved the final manuscript. Dr Shraddha Dalwadi is an attending physician in our department who provided the overall clinical consultation as to the utility of the EVM and the interpretation of the results. She reviewed the basis of the original idea and followed up with review and approval of the final manuscript. Dr. Daniel L Saenz oversaw the work of Dr. Colton Baley as his PhD advisor at the time. Dr. Daniel L Saenz and Dr. Colton Baley both conceived of the original idea. Dr. Daniel L Saenz helped determine the data point elimination criteria and conceived of the statistical analysis details and computations. Dr. Daniel L Saenz wrote the discussion and conclusion sections and also reviewed and approved the manuscript.

## CONFLICT OF INTEREST STATEMENT

The authors declare no conflicts of interest.

## Data Availability

Data will be made available upon reasonable request.

## References

[1] Shamp SJ , Sheikh S , Chang T , et al. Stereotactic body radiotherapy (SBRT) for T2N0 (>3 cm) non‐small cell lung cancer: outcomes and failure patterns. J Radiosurg SBRT. 2021;7(4):271‐277. 34631228 PMC8492054

[2] Aznar MC , Warren S , Hoogeman M , Josipovic M . The impact of technology on the changing practice of lung SBRT. Phys Med. 2018;47:129‐138. doi:10.1016/j.ejmp.2017.12.020 29331227 PMC5883320

[3] Corradetti MN , Mitra N , Bonner Millar LP , et al. A moving target: image guidance for stereotactic body radiation therapy for early‐stage non‐small cell lung cancer. Pract Radiat Oncol. 2013;3(4):307‐315. doi:10.1016/j.prro.2012.10.005 24674403

[4] Sonke JJ , Zijp L , Remeijer P , van Herk M . Respiratory correlated cone beam CT. Med Phys. 2005;32(4):1176‐1186. doi:10.1118/1.1869074 15895601

[5] Yan H , Wang X , Yin W , et al. Extracting respiratory signals from thoracic cone beam CT projections. Phys Med Biol. 2013;58(5):1447‐1464. doi:10.1088/0031-9155/58/5/1447 23399757 PMC6022850

[6] Thengumpallil S , Smith K , Monnin P , Bourhis J , Bochud F , Moeckli R . Difference in performance between 3D and 4D CBCT for lung imaging: a dose and image quality analysis. J Appl Clin Med Phys. 2016;17(6):97‐106. doi:10.1120/jacmp.v17i6.6459 27929485 PMC5690502

[7] Sweeney RA , Seubert B , Stark S , et al. Accuracy and inter‐observer variability of 3D versus 4D cone‐beam CT based image‐guidance in SBRT for lung tumors. Radiat Oncol. 2012;7:81. doi:10.1186/1748-717X-7-81 22682767 PMC3484063

[8] Baley C , Kirby N , Wagner T , et al. On the evaluation of mobile target trajectory between four‐dimensional computer tomography and four‐dimensional cone‐beam computer tomography. J Appl Clin Med Phys. 2021;22(7):198‐207. doi:10.1002/acm2.13310 PMC8292704 34085384

[9] Zhao Y‐q , Gui W‐h , Chen Z‐c , Tang J‐t , Li L‐y . Medical images edge detection based on mathematical morphology. Conf Proc IEEE Eng Med Biol Soc. 2005;2005:6492 ‐6495. doi:10.1109/IEMBS.2005.1615986 17281756

[10] Maini R , Aggarwal H . Study and comparison of various image edge detection techniques. Int J Image Process (IJIP). 2009;3(1):1‐11.

[11] Saif JA , Hammad MH , Alqubati IA . Gradient based image edge detection. Int J Eng Technol. 2016;8(3):153.

[12] Deng G , Cahill LW . An adaptive Gaussian filter for noise reduction and edge detection. In: 1993 IEEE conference record nuclear science symposium and medical imaging conference . IEEE; 1993:1615‐1619.

[13] Li H , Mohan R , Zhu XR . Scatter kernel estimation with an edge‐spread function method for cone‐beam computed tomography imaging. Phys Med Biol. 2008;53(23):6729. 18997269 10.1088/0031-9155/53/23/006

[14] Anam C , Naufal A , Lubis LE , Fujibuchi T . Statistical phase alignment of edge spread function for modulation transfer function measurement on computed tomography images. Physica Medica. 2025;129:104876. 39637629 10.1016/j.ejmp.2024.104876

[15] George G , Oommen RM , Shelly S , Philipose SS , Varghese AM . A survey on various median filtering techniques for removal of impulse noise from digital image. In: 2018 Conference on Emerging Devices and Smart Systems (ICEDSS). IEEE; 2018:235‐238. doi:10.1109/ICEDSS.2018.8544273

[16] Sweeney RA , Seubert B , Stark S , et al. Accuracy and inter‐observer variability of 3D versus 4D cone‐beam CT based image‐guidance in SBRT for lung tumors. Radiat Oncol Lond Engl. 2012;7:81. doi:10.1186/1748-717X-7-81 PMC3484063 22682767

[17] Knybel L , Cvek J , Molenda L , Stieberova N , Feltl D . Analysis of lung tumor motion in a large sample: patterns and factors influencing precise delineation of internal target volume. Int J Radiat Oncol Biol Phys. 2016;96(4):751‐758. doi:10.1016/j.ijrobp.2016.08.008 27788948

